# A solution algorithm for calculating the preload of T-type clamp bolts based on multiphysics coupling

**DOI:** 10.1371/journal.pone.0349093

**Published:** 2026-05-12

**Authors:** Yuening Yang, Yuhang Ou, Rui Lin, Zifan Ding, Han Zhang, Yi Liu

**Affiliations:** 1 College of Electrical Engineering and New Energy, China Three Gorges University, Yichang, Hubei, China; 2 China Energy Consultation (Beijing) Electric Power Research Institute, Beijing, China; 3 State Grid Fujian Electric Power Co., LTD., Sanming, Fujian, China; Henan Polytechnic University, CHINA

## Abstract

Under live-working conditions in distribution networks, the electric, magnetic, and thermal fields generated by current-carrying conductors inevitably affect the contact pressure between the conductor and the connecting fitting, thereby influencing the bolt preload of the fitting. Therefore, this study focuses on evaluating bolt preload in T-type clamps and investigating the influence of current under live-working conditions on that preload. A multiphysics coupling-based approach is proposed for the accurate determination of bolt preload in T-type clamps. First, the governing equations and boundary conditions for the bolt preload of the T-type clamp are derived based on elastic mechanics theory. Then, an iterative coupled electro–magneto–thermal multiphysics model is established, and the governing equations and boundary conditions for bolt preload, accounting for current-carrying effects, are derived. Finally, the preload equations are solved using the finite element method, enabling accurate evaluation of bolt preload under live-working conditions. A comparative analysis between experimental and simulation results is also conducted. Compared with experimental results from the Zhejiang Shangjian Electric Power Testing Institute, the proposed method has an average error of 5.82%. In comparison with methods that neglect multiphysics coupling, the average error is reduced by 11.41%. The proposed method enables an accurate determination of bolt preload in T-type clamps under live-working conditions in distribution networks.

## Introduction

T-type clamps are connecting fittings used to join main conductors in distribution network maintenance operations. To ensure the stable installation of the clamp on the conductor, it is necessary to accurately determine the clamp bolt preload [1,2]. However, with increasing demand for power supply reliability, live-working has gradually replaced outage-based maintenance as the dominant approach in distribution networks [3]. During live working, current-carrying conductors generate additional electric, magnetic, and thermal fields, which affect the contact pressure between the clamp bolts and the conductor [4], thereby leading to variations in bolt preload. Conventional methods for calculating bolt preload under de-energized conditions are no longer applicable to live-working scenarios. Therefore, it is necessary to accurately evaluate the bolt preload of T-type clamps considering multiphysics effects, so as to provide a theoretical basis for the parameter design of distribution network fittings.

Early methods for determining the bolt preload of distribution network clamps mainly relied on indirect and direct experimental measurements [5,6]. In the indirect method, operators use acoustic sensors to estimate bolt preload based on the state equation that relates the variation in pulse-echo time-of-flight to the bolt’s elongation under load [7]. However, with the widespread adoption of live-working operations, continued use of the indirect method requires operators to remain in high-voltage environments for extended periods, leading to a high risk of electric shock [8]. Hence, to ensure operators’ safety, bolt preload measurement methods are shifting toward direct measurement through type testing. In reference [9], the maximum torque of insulation piercing clamp bolts is determined through tensile failure tests of insulated cables, while the minimum torque is determined based on the electrical resistance after cable connection. The bolt preload is then obtained using the conversion relationship between torque and preload. This method avoids exposing measurement personnel to live-working environments during preload determination. However, when applied to different types of clamps, repeated replacement of test specimens is required, resulting in high experimental cost and low efficiency in obtaining bolt preload [10].

With advances in computer hardware, numerical methods have been widely used to determine bolt preload in distribution networks. Reference [11] employed a contact analysis method based on finite element analysis to simulate the relationship between bolt tensile stress and preload under actual external loads. However, because the threaded contact was simplified as an ideal smooth contact surface, the obtained preload was usually underestimated. To address the underestimation of bolt preload in strain clamps, reference [12] decomposed the total torque into thread torque and bearing-surface torque according to the difference in loading characteristics between the bearing surface and the threads. Different friction coefficients were then used to convert these two torque components into preload, thereby reducing the error caused by using a unified empirical friction coefficient. Reference [13] adopted the empirical value of the clamping force of a J-type clamp and, combined with Newton’s first law, obtained the minimum bolt preload required to fasten the clamp onto the conductor, thereby avoiding insufficient preload. These numerical methods are generally established under the assumption that the conductor is de-energized, and only consider the mechanical behavior under the connection state.

However, existing studies have shown that electric and magnetic fields can induce additional loads and alter the stress distribution of structures, while the thermal field further affects contact pressure and connection stability through thermal expansion and thermal stress [14,15]. In addition, the coupled action of electric, magnetic, and thermal fields can significantly change the mechanical response and stability of structures [16–18]. Therefore, the influence of electro–magneto–thermal coupling must be considered under live-working conditions. As a result, existing methods for determining bolt preload are not applicable to live-working conditions, which may lead to inaccurate preload evaluation and cause accidents such as clamp detachment or damage to the conductor core during live-working operations. An accurate theoretical method for determining the bolt preload of distribution network clamps considering multiphysics coupling is urgently needed.

Based on the above analysis, this paper uses the T-type clamp as an example and proposes an electro–magneto–thermal coupling-based model to calculate the bolt preload of line fittings, with the aim of improving the accuracy of bolt preload evaluation under live-working conditions. The main contributions of this paper are summarized as follows:

1) Based on elastic mechanics theory, a fundamental governing equation for the bolt preload of T-type clamps under de-energized conditions is established.
2) Based on multiphysics coupling theory, coupled governing equations for the electric, magnetic, and thermal fields are established to determine the additional contact pressure generated at the interface between the current-carrying conductor and the T-type clamp under multiphysics excitation.
3) The contact pressure induced by multiphysics coupling is introduced into the fundamental governing equation for bolt preload as a correction term, thereby establishing a more accurate model for calculating the bolt preload of T-type clamps under live-working conditions.

The remainder of this paper is organized as follows. The Materials and methods section establishes the governing equation for the bolt preload of fittings under de-energized conditions and further revises the governing equation and boundary conditions based on multiphysics coupling theory. The Results section presents a comparison between experimental and simulation results. The Discussion section concludes the paper and outlines future work.

## Materials and methods

### Mechanical analysis of bolts in T-type clamps

**Fig 1:**
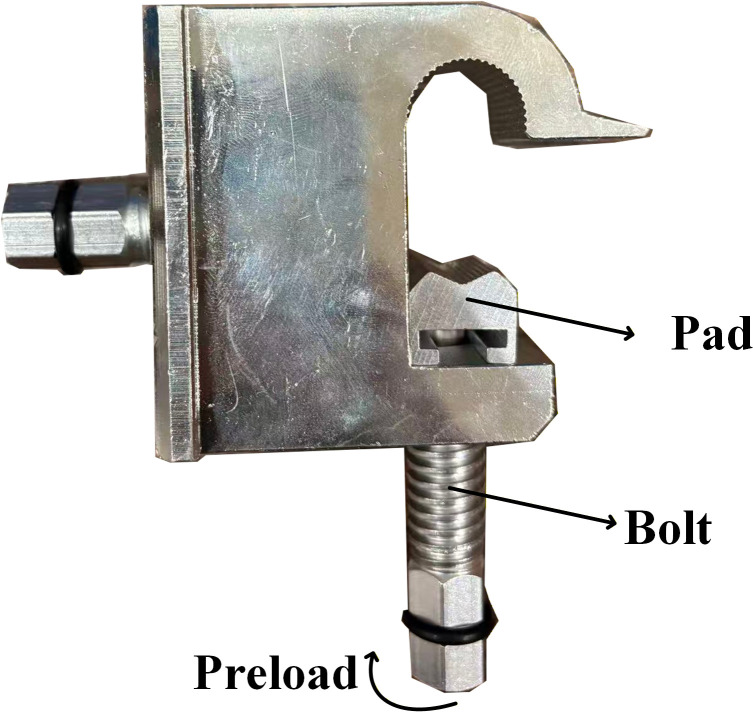
Schematic illustration of the bolt structure of the T-type clamp and the application of preload.

As shown in Fig 1, the T-type clamp is a typical electrical connecting fitting used in 10kV distribution networks. Its main function is to connect the main conductor and the branch conductor, so that the current carried by the conductor can be transmitted and distributed. The main conductor is fixed by the bolt and groove of the T-type clamp.

Bolt preload originates from torsional torque, where the torque direction varies continuously along the thread helix; its analysis can be regarded as a dynamic mechanics problem [19]. Given that the bolt preload is ultimately transmitted as contact pressure at the bolt-main conductor interface, and that this pressure always acts vertically, the mechanical behavior of the bolt–conductor contact interface can first be analyzed. The bolt preload can then be obtained indirectly, thereby transforming the complex dynamic problem into an analysis of static mechanical characteristics.

**Fig 2:**
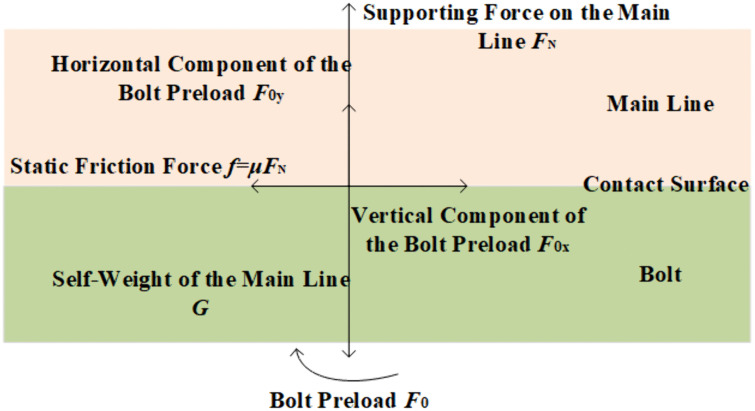
Force analysis of the contact surface between the bolt and the conductor.

**Fig 3:**
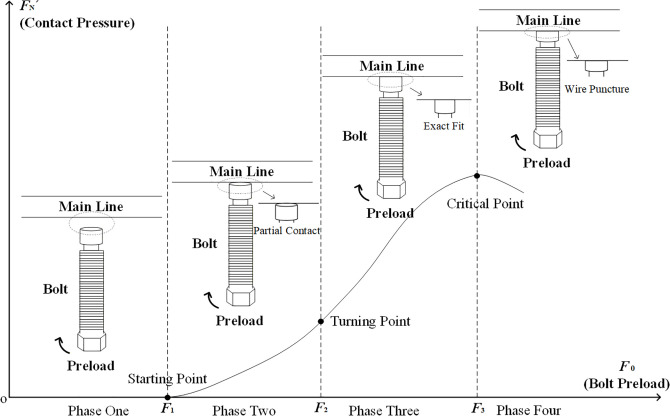
The relationship between bolt preload and contact pressure.

As shown in Fig 2, progressive bolt axial displacement establishes the conductor contact interface. With reference [20] demonstrating interface pressure variation with displacement, the functional relationship between bolt preload and contact pressure can be derived. As shown in Fig 3, upward preload application precedes conductor contact, yielding zero interface pressure *F*_N_’ = 0. At preload *F*_1_, partial contact initiates, with accelerated pressure growth due to rapid interfacial deformation and expanding contact area. At the yield-point preload *F*_2_, conductor curvature reduces the contact area expansion rate, decelerating the pressure increase. The resultant static friction *f* becomes insufficient for retention, causing clamp-conductor slippage and eventual detachment. At the critical preload *F*_3_, full interfacial conformity achieves the threshold pressure, generating adequate static friction for secure clamping. Beyond the critical preload *F*_3_, the bolt penetrates the contact interface and pierces the conductor, resulting in conductor damage. This indicates that, to prevent clamp detachment and conductor damage, it is necessary to accurately determine the bolt preload corresponding to the critical contact pressure.

### Existing calculation methods and deficiencies of clamp bolt preload

Existing methods for calculating the bolt preload of distribution network clamps primarily analyze the contact pressure between the bolt and the conductor, then indirectly determine the bolt preload from it. According to reference [13,21], the governing equation for the bolt preload *F*_0_ of the clamp bolt is given as follows:


$$\left\{ \begin{array}{c} @l@F_{\text{0}}=m{F^{\prime }}_{N-max}\\ {F^{\prime }}_{N-max}={\left[\sigma_{ij}\right]}_{max}A\\ \nabla^{\text{2}}\sigma_{ij}+\frac{\text{1}}{1+\nu }\sigma_{,ij}=0 \end{array}\right.$$ (1)

In this expression, *m* denotes the bolt-preload transmission coefficient, typically assumed to lie between 0.6 and 0.7. *F*_N-max_′ represents the critical contact-surface pressure, and [*σ*_ij_]_max_ is the critical stress of the contact surface. *A* is the effective loaded area at the interface between the main conductor and the bolt. ∇^2^*σ*_ij_ indicates the Laplacian applied to the contact-surface stress tensor, while *σ*,_ij_ denotes the second-order partial derivatives of that stress tensor. *v* represents the Poisson’s ratio of the contact interface material [22]. Here, the stress field characterizes the distribution of bolt-preload-induced pressure per unit area on the contact surface, and can be directly converted to contact pressure.

Conventional distribution network maintenance and retrofit operations are usually carried out under de-energized conditions. Accordingly, equation (1) is established under the assumption that the conductor is not energized and only considers the mechanical behavior under the connection state. This assumption is applicable only to de-energized operating scenarios. However, with the widespread adoption of live-working in distribution networks, a large proportion of maintenance work is now performed in a 10 kV high-voltage environment. Under such conditions, the electric, magnetic, and thermal fields generated by the current-carrying conductor will change the contact pressure between the bolts of the T-type clamp and the conductor, and the conventional preload model based on the de-energized assumption cannot accurately reflect the actual operating condition.

Therefore, based on the conventional preload model established under the de-energized assumption, this study first calculates the pressures induced by the electric, magnetic, and thermal fields of the current-carrying conductor in the uncoupled state according to Maxwell’s equations, and uses them to perform the first correction of the preload model. Then, considering the interactions among multiple physical fields [23], a multiphysics coupling theory is introduced to establish the governing equations for the coupled fields. After the iterative convergence criterion is defined, the combined pressure generated by the coupled multiphysics fields is solved and used to perform the second correction of the preload model. Finally, the accurate bolt preload of the T-type clamp under live-working conditions in distribution networks is obtained.

### Bolt preload of the T-type clamp under the individual action of multiple physical fields

When the main conductor of a distribution line is in the current-carrying state, an alternating current exists inside the conductor. The alternating current density ***J***_***c***_ inside the conductor is produced by the directed motion of charge carriers driven by the alternating electric field ***E***, whose direction and magnitude vary in sync with the current. According to Ohm’s law, the relationship between ***J***_***c***_ and ***E*** is:


$$E=J_{c}/\sigma^{\prime }$$ (2)

In the equation, *σ*′ denotes the electrical conductivity of the feeder conductor.

**Fig 4:**
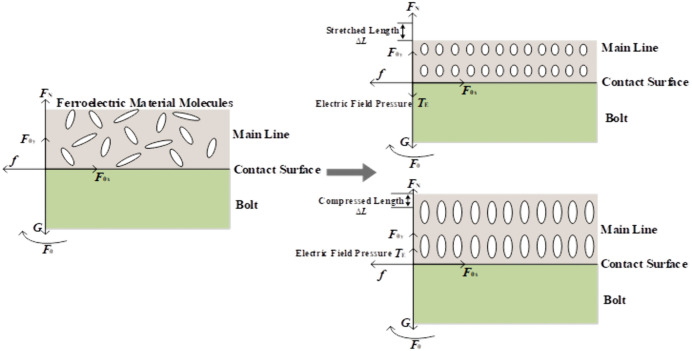
Alternating electric field–induced electrostriction at the conductor–bolt contact surface.

As shown in Fig 4, the electric field induces an electrostriction effect at the contact interface. [24]

According to Maxwell’s stress‐tensor theory, the electric‐field pressure *T*_E_ produced by the electrostrictive effect is given by:


$$T_{\text{E}}=\frac{\text{1}}{\text{2}}\varepsilon_{\text{0}}{\left|E\right|}^{\text{2}}⬝A$$ (3)

*ε*₀ denotes the relative permittivity of the contact interface, and *A* is the effective area over which the contact interface is subjected to force.

In an alternating electric field, directed displacement of charges within the feeder conductor gives rise to a conduction current density ***J***_c_. According to Ampère’s law in Maxwell’s equations [25], the distribution of the magnetic field intensity ***H*** around the current-carrying conductor can be determined as follows:


$$\nabla \times H=\frac{\partial D}{\partial t}+J_{\text{c}}$$ (4)

***H*** is the magnetic field strength induced by the alternating electric field, where B=μH, *μ* is the absolute permeability of the feeder-conductor material, and ***B*** is the corresponding magnetic flux density, *t* is the time variable. ***D*** is the elec*t*ric displacement at the conductor–bolt contact interface due to the alternating current within the feeder conductor, where D=εE, *ε* is the absolute permittivity of the feeder-conductor material.

**Fig 5:**
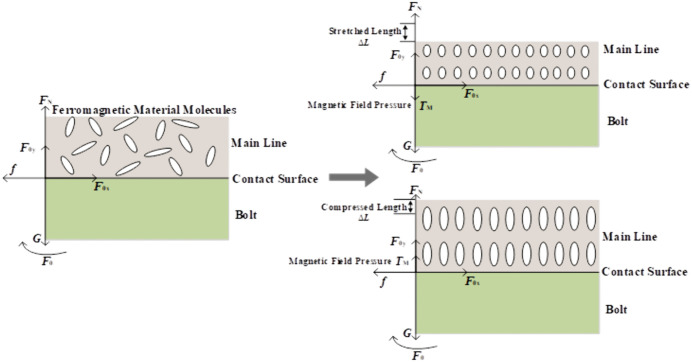
Magnetostrictive effect at the conductor–bolt contact interface induced by the alternating magnetic field.

As shown in Fig 5, the periodic reversal of the magnetic field direction causes the magnetic domains within the ferromagnetic material at the conductor–bolt contact interface to repeatedly reorient, inducing lattice distortions in the material’s molecules and thereby generating a magnetostrictive effect [26].

According to magnetoelastic theory, the effect of the magnetic field can be equivalently represented as a magnetic force *T*_M_ acting on the contact interface, which is expressed as:


$$T_{\text{M}}=\frac{\text{1}}{\text{2}}\mu {\left|H\right|}^{\text{2}}⬝A$$ (5)

**Fig 6:**
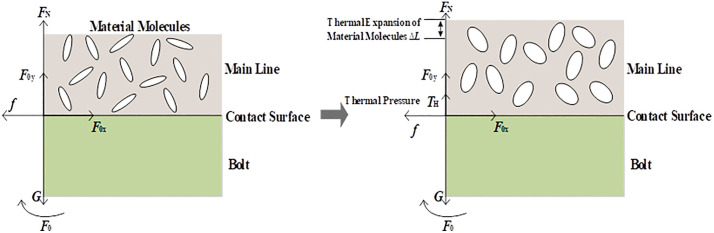
Thermal pressure at the conductor–bolt contact interface induced by the temperature field.

As shown in Fig 6, when an alternating current flows through the main conductor, collisions between electrons and the material’s lattice convert the electrons’ kinetic energy into heat, raising the temperature at the conductor–bolt contact interface and producing a Joule heating effect. As the contact-interface temperature increases, the thermal vibrations of the material’s molecules intensify, and their average separation grows, resulting in thermal expansion [27].

Since the main conductor in the T-type clamp is fixed within the main-conductor groove by the bolts, the thermal expansion at the contact interface is constrained, thereby generating an elastic reaction force, namely thermal pressure. In addition, because convective heat transfer exists between the main conductor and the surrounding air in the T-type clamp, the balance between heat generation caused by alternating current and heat dissipation should also be considered [28].


$$I^{\text{2}}R=hA^{\prime }\vartheta$$ (6)

*I* is the current through the main conductor, *R* is the conductor’s average electrical resistance, *h* is the convective heat transfer coefficient, and *A*′ is the heat-dissipating surface area of the main conductor. *ϑ* denotes the temperature difference between the upper surface of the main conductor and the bolt–conductor contact interface.

After the temperature difference *ϑ* is obtained from equation (6), the thermal field-induced force *T*_H_ generated by thermal expansion in Fig 6 can be derived from thermoelastic theory [29] as follows:


$$T_{H}=E_{t}\left(\frac{\Delta \delta }{L_{\text{0}}}-k⬝\vartheta \right)⬝A$$ (7)

In the equation above, *E*_t_ is the elastic modulus of the main conductor, Δ*δ* is the permissible thermal expansion of the main conductor, *L*_0_ is the initial thickness of the main conductor, and *k* is the thermal conductivity coefficient.

In summary, the electric, magnetic, and thermal fields all generate additional forces, namely *T*_E_, *T*_M_, and *T*_H_, at the contact interface between the main conductor and the bolt. Therefore, based on the bolt preload *F*_0_ obtained from the mechanical analysis, equations (3), (5), and (7) are further substituted into equation (1) to obtain the compensated preload *F*_0_′. By superposing *F*_0_ and *F*_0_′, the bolt preload *F*_0cp_ of the T-type clamp under multiphysics fields can be obtained.


$$F_{0cp}=F_{\text{0}}+{F^{\prime }}_{\text{0}}=m\text{(}{F^{\prime }}_{N-max}+T_{\text{Emax}}+T_{\text{Mmax}}+T_{Hmax}\text{)}$$ (8)

*T*_Emax_, *T*_Mmax_ and *T*_Hmax_ are, respectively, the maximum values of the additional contact pressures due to the electric, magnetic, and thermal field at the interface between the main conductor and the bolt.

### Multiphysics coupling calculation model for the bolt preload of the T-type clamp

Coupling interactions exist among the electric, magnetic, and thermal fields. The electric field—directly excited by the current—serves as the primary source, producing a varying magnetic field as an intermediate physical field. The magnetic field influences the temperature field via the magnetothermal effect, while its strength is diminished by the demagnetizing effect at elevated temperatures; however, as an intermediate field, its feedback on both the electric and temperature fields is negligible. The temperature field—driven by environmental heat exchange—reduces the contact resistance, thereby enhancing the current’s thermal effects, which in turn significantly react back on the electric field; this coupling cannot be ignored. Based on the nature of the excitation sources and the causal relationships among the fields, the iterative coupling sequence is as follows: electric field → magnetic field → temperature field → electric field.

It follows that, for the coupling between the electric and magnetic fields, only the effect of the electric field on the magnetic field is considered. In the electric field, the conduction current density ***J***_c_ and the displacement current density ***J***_w_ together form the closed loops of the magnetic field. According to Ohm’s law and Maxwell’s equations, the relationships between ***J***_c_ and ***J***_w_ and the electric field ***E*** are:


$$\left\{ \begin{array}{c} @l@J_{\text{c}}=-\sigma^{\prime }\nabla \Phi \\ J_{\text{w}}=-\varepsilon \nabla \frac{\partial \Phi }{\partial t} \end{array}\right.$$ (9)

In the equation, *Φ* denotes the electric potential between the upper surface of the main conductor and the contact interface between the main conductor and the bolt.

By substituting equation (9) into equation (4), the governing equation describing the influence of the electric field on the magnetic field can be obtained as follows:


$$\nabla \times H=-\sigma^{\prime }\left(\Theta \right)\nabla \Phi -\varepsilon \left(\Theta \right)\nabla \left(\frac{\partial \Phi }{\partial t}\right)$$ (10)

For the coupling between the magnetic and temperature fields, only the effect of the thermal field on the magnetic field is considered. When the magnetic domains in Fig 5 continuously flip and raise the contact-surface temperature to the Curie temperature, the thermal expansion of the magnetic medium directly disrupts the ordered domain structure, causing a precipitous drop in the magnetic flux density ***B*** and thus altering the magnetic field intensity ***H***. The equivalent magnetic permeability *μ* (*Θ*) based on the Curie–Weiss relation is expressed as:


$$\mu \left(\Theta \right)=\mu_{\text{0}}\left[1+\frac{C}{\Theta -\Theta_{c}}\;\right]\left(\Theta <\Theta_{c}\right)$$ (11)

In the equation, *C* is the Curie constant of the contact-surface material, and *Θ*_c_ is its Curie temperature; both parameters can be measured directly by experiment and treated as known quantities. *Θ* denotes the temperature at the contact interface. *μ*_0_ denotes the magnetic permeability of vacuum and is independent of temperature.

By combining equation (11), the thermal-corrected magnetic field relation can be obtained as follows:


$$B=\mu_{\text{0}}\left[1+\frac{C}{\Theta -\Theta_{c}}\right]\cdot H$$ (12)

The coupling between the thermal field and the electric field requires consideration of bidirectional effects. The alternating current generates heat due to the surface resistance of the main conductor, creating a temperature difference *ϑ* between the upper surface of the conductor and the conductor–bolt contact interface. Electrons diffuse from the high‐temperature end to the low‐temperature end, causing charge accumulation and generating an electric field intensity ***E*** opposite to the temperature gradient, thereby altering the potential distribution *Φ* between the conductor’s upper surface and the contact interface. According to the Seebeck effect, a temperature gradient induces a thermoelectric potential [30,31]. Therefore, the expressions for the electric field and current density are no longer accurate in the coupled thermal–electric field and should be corrected as follows:


$$\left\{ \begin{array}{c} @l@E=-\nabla \Phi +\alpha_{s}\nabla \Theta \\ J_{\text{c}}=\sigma^{\prime }E=\sigma^{\prime }\left(-\nabla \Phi +\alpha_{s}\nabla \Theta \right) \end{array}\right.$$ (13)

𝛼_𝑠_ denotes the Seebeck coefficient at the contact interface, which can be directly measured experimentally and treated as a known parameter.

Similarly, when the conduction current density ***J***_c_ and the displacement current density ***J***_w_ of the alternating current flow through the main conductor under the driving action of the electric field intensity ***E***, Joule heating due to the conductor’s surface resistance causes a local temperature rise. The heat flux density *κ* then diffuses from the high-temperature region to the low-temperature region, creating a temperature difference *ϑ*. According to Fourier’s law and the law of conservation of energy, the generated Joule heat is equal in magnitude to the heat exchanged with the environment. Therefore, the governing equation for the coupling between the thermal field and the electric field is:


$$\left\{ \begin{array}{c} @l@\rho c_{p}\frac{\partial \Theta }{\partial t}=\nabla \cdot \left(k\nabla \Theta \right)+Q\\ Q=\sigma^{\prime }{\left|E\right|}^{\text{2}}=\sigma^{\prime }{\left(-\nabla \Phi +\alpha_{s}\nabla \Theta \right)}^{\text{2}} \end{array}\right.$$ (14)

*ρ*is the material density of the main conductor, *c*_p_ is its specific heat capacity, *k* is its thermal conductivity, *T* is the thermal field, and *Q* is the heat generated by the current’s thermal effect.

In summary, the governing equations for the multi-field coupling are:


$$\left\{ \begin{array}{c} @l@\nabla \times H=-\sigma^{\prime }\left(\Theta \right)\nabla \Phi -\varepsilon \left(\Theta \right)\nabla \left(\frac{\partial \Phi }{\partial t_{\text{1}}}\right)\\ B=\mu_{\text{0}}\left[1+\frac{C}{\Theta -\Theta_{c}}\right]\cdot H\\ \rho c_{p}\frac{\partial \Theta }{\partial t}=\nabla \cdot \left(k\nabla \Theta \right)+Q\\ Q=\sigma^{\prime }{\left|E\right|}^{\text{2}}=\sigma^{\prime }{\left(-\nabla \Phi +\alpha_{s}\nabla \Theta \right)}^{\text{2}} \end{array}\right.$$ (15)

After *h* iterative cycles, the multi-field iterative coupling equation can be expressed as:


$$\left\{ \begin{array}{c} @l@\nabla \times H^{\left(h+1\right)}=-\sigma^{\prime }\left(\Theta^{\left(h\right)}\right)\nabla \Phi^{\left(h\right)}\\ -\varepsilon \left(\Theta^{\left(h\right)}\right)\nabla \left(\frac{\partial \Phi^{\left(h\right)}}{\partial t_{\text{1}}}\right)\\ B^{\left(h+1\right)}=\mu_{\text{0}}\left[1+\frac{C}{\Theta^{\left(h\right)}-\Theta_{c}}\right]\cdot H^{\left(h\right)}\\ \rho c_{p}\frac{\partial \Theta^{\left(h+1\right)}}{\partial t}=\nabla \cdot \left(k\nabla \Theta^{\left(h\right)}\right)+\sigma^{\prime }{\left|\nabla \Phi^{\left(h\right)}\right|}^{\text{2}}\\ E^{\left(h+1\right)}=-\nabla \Phi^{\left(h\right)}+\alpha_{s}\nabla \Theta^{\left(h\right)}\\ \nabla \Phi^{\left(h+1\right)}=-E^{\left(h+1\right)} \end{array}\right.$$ (16)

In the above equation, the computation sequence must strictly follow the order of the formulas, and the output variables include the magnetic flux density ***B***, the electric field intensity ***E***, and the contact-surface temperature *Θ*.

The multiphysics iteration is considered to have reached equilibrium when the order of magnitude of the iteration residuals between two consecutive steps for all physical fields is less than 10^−5^. The convergence criterion is defined as [32]:


$$w^{\left(h\right)}\text{\textbackslash{}hspace\{0.33em}\}=\text{\textbackslash{}hspace\{0.33em}\}max\left(\frac{∥E^{\left(h+1\right)}-E^{\left(h\right)}∥}{∥E^{\left(h+1\right)}∥},\text{\textbackslash{}hspace\{0.33em}\}\frac{∥B^{\left(h+1\right)}-B^{\left(h\right)}∥}{∥B^{\left(h+1\right)}∥},\text{\textbackslash{}hspace\{0.33em}\}\frac{∥\Theta^{\left(h+1\right)}-\Theta^{\left(h\right)}∥}{∥\Theta^{\left(h+1\right)}∥}\right)$$ (17)

where *w*^*(h)*^ is the iteration residual at the *h*-th step. The iteration terminates when *w*^*(h)*^ < 10 ⁻ ⁵, outputting the magnetic flux density *B*^*(h)*^, electric field intensity *E*^*(h)*^, and contact interface temperature *Θ*^*(h)*^. If the convergence condition is not met, the computation returns to equation (17) for the (*h* + 1)-th iteration until the condition is satisfied.

If the multiphysics iterative coupling governing equations converge at the *h*_0_-th iteration, the converged electric field, magnetic field, and thermal field are substituted into equation (3), (5), and (7). Accordingly, the pressure *F*_0_′ in equation (8), which is obtained under the independent action of multiple physical fields, is further corrected to the coupled multiphysics pressure *F*_0_″. The bolt preload *F′*_0cp_ under multiphysics iterative coupling can then be expressed as:


$${F^{\prime }}_{0cp}=F_{\text{0}}+{F^{\prime \prime }}_{\text{0}}$$ (18)

This study did not involve human participants or animals. Therefore, ethical approval was not required.

## Results

### Experimental measurement of the bolt preload of the T-type clamp and validation of the proposed calculation method

#### Experimental setup.

**Fig 7:**
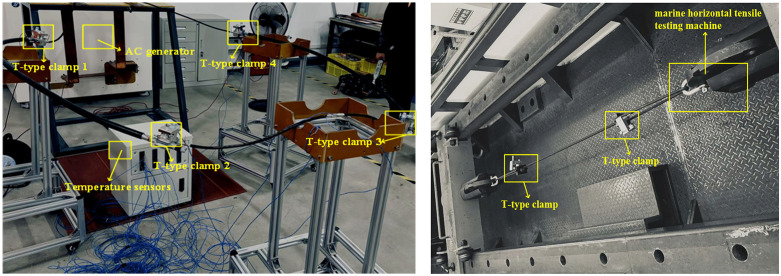
On-site layout of the experiment for the T-type clamp. (a) Setup for the temperature-rise experiment. (b) Setup for the displacement experiment.

To verify the accuracy of the proposed algorithm, experiments were conducted at the Zhejiang Shangjian Electric Power Testing Institution, as illustrated in Fig 7. When a horizontal tensile testing machine applies kN-level tension for displacement measurement, excessive heating due to conductor current flow can easily lead to hazardous incidents such as conductor breakage, posing a serious safety threat to both equipment and personnel. Therefore, conducting sequential temperature-rise and displacement tests represented the optimal scheme achievable under guaranteed safety conditions.

For both tests, a 240 mm² main conductor and a 70 mm² branch conductor were used, and TLL10-2-240/70^2^ T-type clamps were chosen as the test specimens. According to the Chinese National Standard GB/T 18857‑2019 “Technical Guide for Live Working on Distribution Lines” [33], live working on distribution networks should be conducted under favorable weather conditions. Therefore, in this study, the ambient temperature was assumed to be 25°C and was maintained by the laboratory’s central air-conditioning system. The temperature-rise test simulated the electromagnetic and thermal excitation from the current-carrying conductor to the clamp, using a ZYT AC generator (0–500 A output range) as the power source. The displacement test replicated the mechanical loading on the clamp and measured its displacement, employing a 3YMW-500C-12m marine horizontal tensile testing machine and a torque tester.

**Fig 8:**
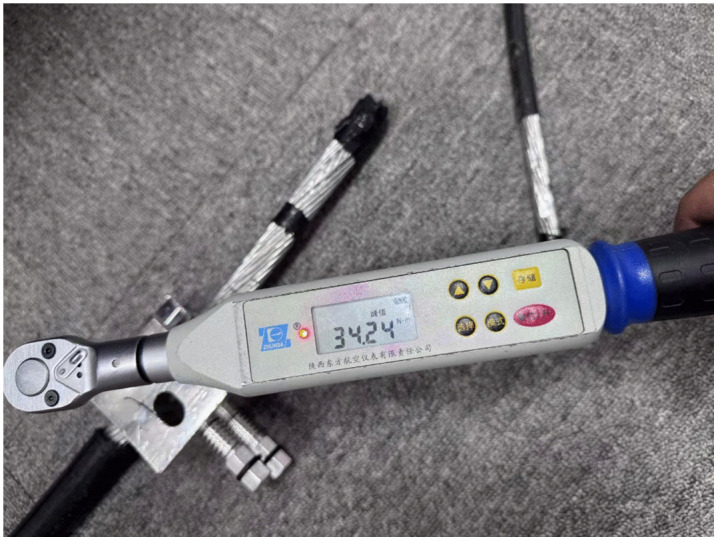
Application of torque to the T-type clamp using a torque wrench.

To avoid random errors from a single clamp specimen, the research team selected four T-type clamps connected in series via the main and branch conductors. The four test clamps were positioned at different locations along the main conductor, with the end conductors connected to the positive and negative terminals of the ZYT AC generator, as shown in Fig 8-a. The laboratory ambient temperature was held constant at 25 °C. Under normal grid operation, the alternating current amplitude ranged from 100 A to 340 A, and could reach up to 400 A under extreme conditions. Therefore, the conductor current amplitude was chosen between 100 A and 400 A. In the temperature-rise test, once the clamp temperature stabilized, the still-hot clamp was immediately mounted on the marine horizontal tensile machine for the displacement test. This procedure both avoided the safety hazard of energizing the tensile machine and effectively simulated simultaneous electromagnetic, thermal, and mechanical loading.

For the displacement test, a torque wrench was used to apply preload to the clamp bolts. According to the Chinese National Standard GB/T 2317 “Test Methods for Power Fittings” [34] for 240 mm² conductors, the applied torque was 34 N·m to 42 N·m, with measurement increments of 0.5 N·m. The specific procedure for applying torque to the T-type clamps is shown in Fig 8.

#### Experimental procedure.

In the control area, the power supply was switched on to apply currents of 100 A, 150 A, 200 A, 250 A, 300 A, 350 A, and 400 A to the main conductor and the four test T-type clamps, as shown in Fig 7-a. Once the T-type clamp temperature reached equilibrium, the uncooled clamp was directly mounted on the shipboard horizontal tensile testing machine for displacement testing. This approach avoided the safety hazards associated with energizing the testing machine while effectively simulated the simultaneous application of the electric, magnetic, and thermal fields, as well as the conductor tension. As shown in Table 1, the time interval between the end of the temperature-rise test and the start of the displacement test was less than 3 minutes. The resulting temperature drop of the fitting was only 2.59–9.62°C. This variation represents a minor perturbation relative to the significant temperature rise of 40.5–85.62°C observed during the temperature-rise test, and the associated experimental error was considered acceptable.

**Table 1:** Average Temperature at the Conductor-Fitting Contact Interface (°C).

Applied Current	Temperature in Temperature Rise Test	Temperature in Stress Test	Temperature Difference (3-min Interval)
**400A**	85.62	76.00	9.62
**350A**	78.55	70.62	7.93
**300A**	70.34	63.73	6.61
**250A**	63.54	57.70	5.84
**200A**	55.72	50.81	4.91
**150A**	47.80	43.93	3.87
**100A**	40.50	37.91	2.59

During the displacement test, a torque wrench (shown in Fig 8) was used to apply torque to the T-type clamp, starting from 42 N ∙ m and gradually decreasing to 34 N ∙ m in 0.5 N ∙ m steps. The clamp was then mounted on the YMW-500C-12m shipboard horizontal tensile testing machine (Fig 8). A tension of 1.14 kN was applied to the conductor, and the displacement of each T-type clamp was recorded over a duration of 1 minute.

**Fig 9:**
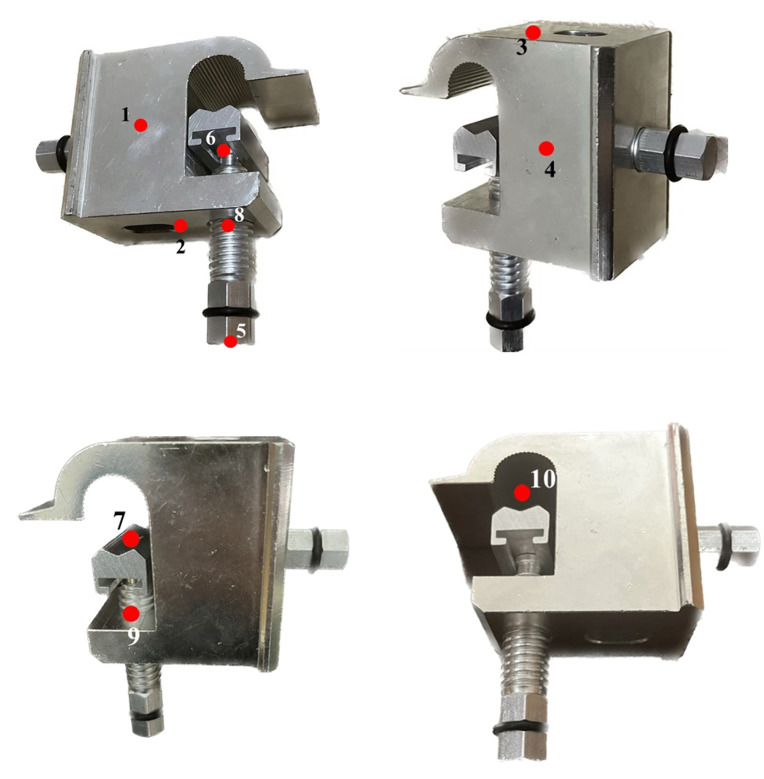
Application of torque to the T-type clamp using a torque wrench.

The selection of 10 displacement measurement points shown in Fig 9 was aimed at efficiently and accurately capturing the overall displacement of key regions of the T-type clamp under load, using a limited yet representative set of locations. The displacement distribution across each face of the T-type clamp is continuous and smooth, with points on the same face exhibiting similar displacement direction and magnitude. Dense placement of points over the entire surface would generate substantial redundant data. Therefore, the geometric center point of each face was selected as its representative.

To validate that the selected geometric center points adequately represent their respective faces, the displacement data of all nodes on each face from the finite element model were extracted and analyzed. As shown in Table 2, for all 10 clamp surfaces, the deviation between the displacement at the geometric center point and the average displacement of all nodes on that face is less than 2%. Furthermore, the deviation between the maximum displacement and the average displacement on any given face is within 5%. The small deviation between the geometric center point displacement and the face average displacement confirms that it can effectively represent the overall displacement state of that face. Consequently, selecting the geometric center points allows the displacement trend of the entire clamp to be represented with a minimal number of measurement points.

**Table 2:** Displacement Values on Clamp Surfaces(m).

Surface S*ᵢ*	Displacement at Geometric Center	Average Displacement of All Nodes	Maximum Displacement
**S**_**1**_	1.3788 × 10^−5^	1.3734 × 10^−5^	1.4555 × 10^−5^
**S**_**2**_	1.7518 × 10^−5^	1.7776 × 10^−5^	1.8487 × 10^−5^
**S**_**3**_	8.2176 × 10^−6^	8.1158 × 10^−6^	8.6745 × 10^−6^
**S**_**4**_	1.3165 × 10^−5^	1.3362 × 10^−5^	1.3896 × 10^−5^
**S**_**5**_	2.3642 × 10^−5^	2.3505 × 10^−5^	2.4957 × 10^−5^
**S**_**6**_	8.3183 × 10^−6^	8.1595 × 10^−6^	8.7808 × 10^−6^
**S**_**7**_	8.5469 × 10^−6^	8.5871 × 10^−6^	9.0221 × 10^−6^
**S**_**8**_	4.3257 × 10^−9^	4.3525 × 10^−9^	4.5662 × 10^−9^
**S**_**9**_	4.4126 × 10^−9^	4.3539 × 10^−9^	4.6579 × 10^−9^
**S**_**10**_	9.1469 × 10^−6^	9.2429 × 10^−6^	9.6555 × 10^−6^

**Fig 10:**
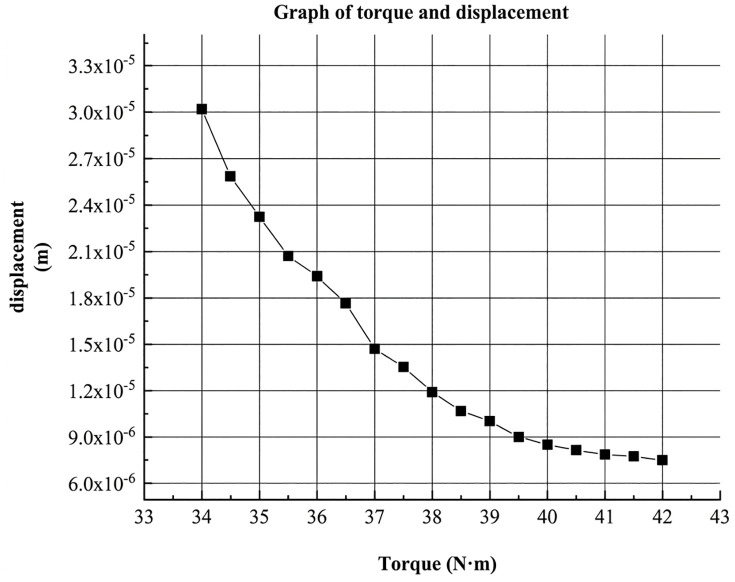
Variation Curve of the Average Displacement of the T-Type Clamp under Different Applied Torques.

Under applied torques ranging from 34.5 N·m to 42 N·m, the average displacement of the T-type clamp at each torque level was measured. The resulting variation curve is shown in Fig 10.

The curve shows that after the torque reaches 41 N·m, it levels off, indicating that 41 N·m is close to the critical point identified in Figs 2-3. The average displacement of the clamp under torques between 41 N·m and 42 N·m all meet the standard, remaining below the order of 10 ⁻ ⁶. Further non-destructive testing within this torque range revealed conductor damage at 41.5 N·m. Therefore, 41 N·m is selected as the optimal measurement torque. Substituting the average displacement into Equation (19) yields the stress at the contact interface between the conductor and the bolt:


$$\sigma_{ij}=l_{m}(\frac{\varepsilon_{\text{0}}}{\text{2}}{\left|E\right|}^{\text{2}}+\frac{\varepsilon_{\text{0}}}{\text{2}}{\left|B\right|}^{\text{2}})+\frac{l_{m}E_{t}(\alpha_{s}-k)}{L_{\text{0}}}u_{z}$$ (19)

Where *l*_*m*_ is the Lamé constant, ***u*** is the displacement at any point on the contact surface, *u*_*z*_ is the longitudinal displacement component along the z-axis. Using Equation (1), this corresponds to an optimal preload of 1.85 kN.

**Fig 11:**
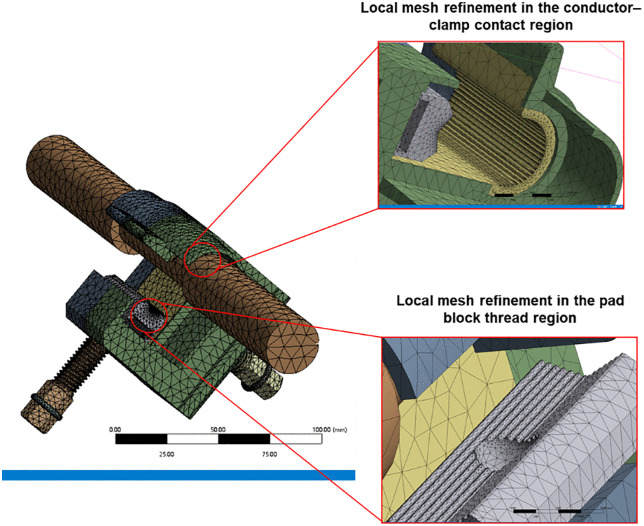
Schematic illustration of mesh generation and local mesh refinement.

During the simulation, the ambient temperature was assumed to be the same as that in the experiment, namely 25 °C. Under this condition, the surrounding environment was taken as natural convection. Accordingly, the parameter values used for preload calculation are listed in Table 3. The values in the table are standard engineering values at room temperature for aluminum (fitting material) and copper (conductor core material) [35], and are applicable to the conditions considered in this study. The fitting model has a height of 73 mm, a length of 62 mm, and a width of 50 mm. The pad block beneath the clamp is 18 mm wide. The bolt has a total length of 75 mm and a diameter of 14 mm. The conductor used in the model matches this fitting, with a core cross-sectional area of 240 mm^2^. This simulation involves electro–magneto–thermal coupling, and the final quantities of interest are the bolt preload and the contact mechanical response of the T-type clamp assembly. Therefore, during mesh generation, the physics preference was set to mechanical so that the generated mesh would be more suitable for structural and contact analysis. Considering that the main conductor–clamp contact region and the bolt thread region have relatively small geometric features and are more sensitive to stress transfer and contact-state variation, local mesh refinement was applied to these key regions, as shown in Fig 11, to improve local discretization accuracy and ensure smooth mesh transition. After meshing, the global mesh size was 6.47 mm, with a total of 278,332 nodes and 174,245 elements. According to the actual installation condition of the clamp, the back surface of the clamp was defined as a fixed support to constrain its displacement degrees of freedom, and fixed supports were also applied at both ends of the main conductor. The main conductor–clamp contact interface and the clamp–bolt contact interface were both defined as No Separation to simulate the contact and force-transfer characteristics in the actual connection process. Consistent with the experiment, the current applied to the main conductor ranged from 100 to 400 A, with an increment of 50 A, and was applied along the axial direction of the main conductor. The applied bolt torque ranged from 34 to 42 N·m, with an increment of 0.5 N·m, to simulate the bolt preloading process. The above data are available in S1 Dataset.

**Table 3:** Parameter Settings for the T-type Clamp and Conductor.

	Measurement	T-type Clamp	Conductor
**Electric Field**	Conductivity(MS·m^-1^)	30.30	59.98
**Magnetic Field**	Permeability(H/m)	300.23	1.01
**Thermal Field**	Thermal Conductivity(W/(m ∙ K))	201	400
	Specific Heat Capacity at Constant Pressure(J/(kg ∙ K))	900	385
	Convection Coefficient(W/(m^2^ ∙ K))	5	5
	Surface Emissivity	0.981	0.972
**Material Properties**	Density(kg ∙ m^-3^)	2700	8940
	Microhardness (GPa)	3	1.9
	Mean Slope of Roughness	0.41	0.42
	Mean Roughness Height(μm)	0.998	1.001
	Young’s Modulus(GPa)	69	126
	Poisson’s Ratio	0.33	0.34

**Fig 12:**
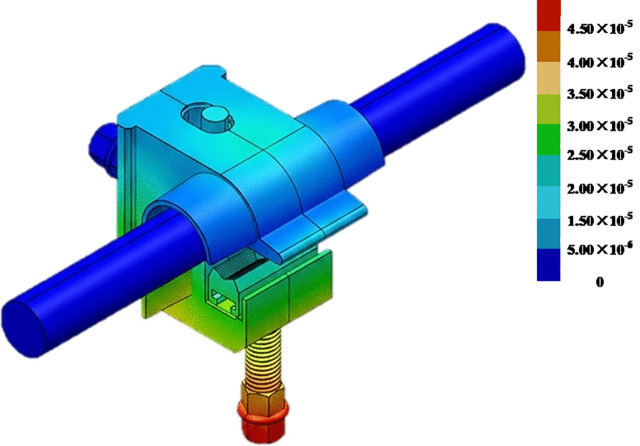
Displacement distribution of the T-type clamp calculated using the conventional algorithm (unit: m).

At 250 A, the displacement results obtained by the conventional algorithm without considering multiphysical-field coupling are shown in Fig 12. Displacements at measurement points 1–10 were recorded sequentially, and the average displacement of these ten points was used to calculate the stress via Equation (1). From this, the bolt preload was back-calculated to be 1.09 kN. The same procedure was then applied to the remaining current levels to derive the corresponding bolt preload values.

**Fig 13:**
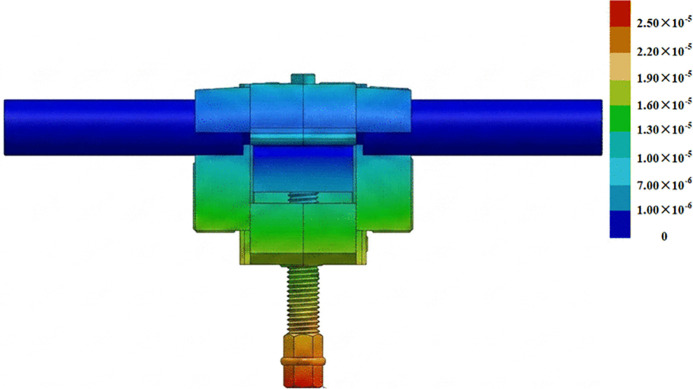
Displacement distribution of the T-type clamp calculated using the algorithm presented in this paper (unit: m).

When the current was 400 A, the displacement results obtained using the proposed multi-field coupling algorithm for determining the bolt preload of the T-type clamp are shown in Fig 13. Displacements at measurement points 1–10 were recorded sequentially, and the average displacement was used to compute the stress via Equation (18), from which the bolt preload was back-calculated as 1.73 kN. The same procedure was then applied to the remaining current levels to derive their corresponding bolt preload values.

**Fig 14:**
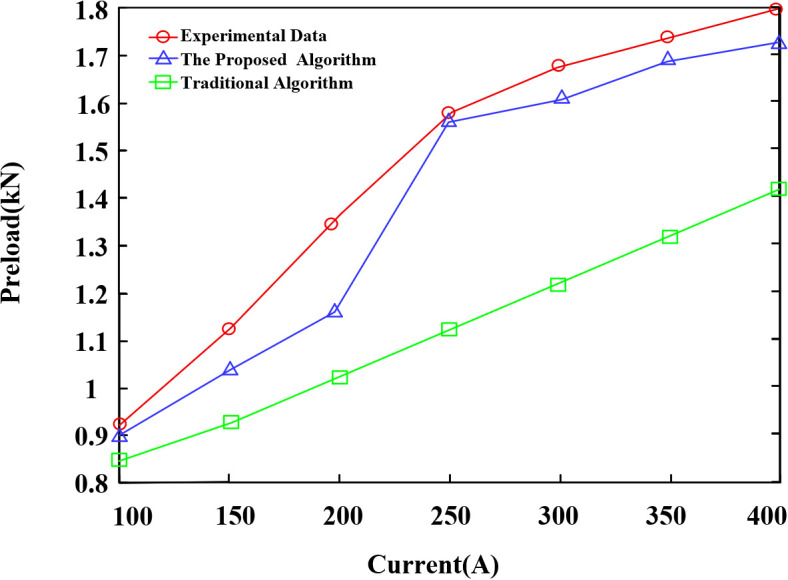
Bolt preload obtained by various methods.

As shown in Fig 14, within the 150–200 A current range, the correction effect of the multiphysics coupling model is not yet fully evident. This is because, at relatively low current levels, the electric field and the induced magnetic field are weak, and the additional contact pressure caused by electrostriction and magnetostriction is relatively small in the calculation. As a result, the advantage of introducing the multiphysics model in correcting the calculation result is limited, and the difference between the proposed method and the conventional method remains small. Additionally, due to experimental constraints, the temperature rise test and the displacement test were conducted sequentially. This approach inevitably introduced a cooling period for the fitting between the two tests. Within the 150–200 A range, the fitting experienced a certain temperature rise. Because the temperature increase was still small at this stage, the attenuation of thermoelastic stress caused by the cooling process became more sensitive and pronounced. Combined with the limited correction advantage of the multiphysics coupling model mentioned above, this further enlarged the error between the calculated and experimental results in this current range, making it significantly higher than that at other current levels.

The results show that the average difference between the calculated values of the proposed algorithm and the measured values under the seven current levels is 0.078 kN, with an average error of 5.82%. For the conventional algorithm, the average difference between the calculated and measured values is 0.261 kN, with an average error of 17.23%. Compared with the conventional algorithm, the proposed algorithm reduces the average difference by 0.183 kN and the average error by 11.41%. The trend in variation of the proposed algorithm is generally consistent with that of the measured data across all current levels. In contrast, the calculation method that does not account for conductor current is approximately linear and monotonic, and it cannot characterize the influence of multiphysics fields on the bolt preload of the T-type clamp.

## Discussion

This paper proposes a bolt preload algorithm for fittings based on multiphysics coupling. The experimental and simulation results reveal that:

1) Based on multiphysics coupling theory, the iterative coupling sequence and convergence criterion among the electric, magnetic, and thermal fields of the current-carrying conductor are explicitly defined, and the governing equation for the bolt preload of the T-type clamp on the current-carrying conductor is re-derived, making it applicable to bolt preload calculation under live-working conditions.
2) By comparison with the bolt preload experiments conducted by Zhejiang Shangjian Electric Power Testing Institution, the average error between the bolt preload calculated by the proposed multiphysics coupling method and the measured value is 5.82%. Compared with the conventional bolt preload calculation method, the accuracy is improved by 11.41%.
3) This calculation theory can provide more accurate bolt preload values for fittings, thereby reducing engineering problems such as fitting slippage caused by insufficient bolt preload and conductor-core damage caused by excessive bolt preload during live-working operations. It also provides a theoretical basis for the subsequent improved design of fittings.

In summary, the proposed calculation method can more accurately solve for the bolt preload of fittings under live working conditions. Future work will primarily focus on considering the effects of factors such as corona corrosion, material aging, and bolt relaxation on the bolt preload to construct a more precise calculation model.

## Supporting information

S1 Dataset Minimal anonymized dataset. (XLS)

## References

[1] Li N, Hu JL, Zhan XX. Reliability assessment of elastic distribution network based on fault correlation matrix. J Xi’an Polytech Univ. 2024;38(1):1–8.

[2] Liu J, Zhou Y, Li Y, Lin G, Zu W, Cao Y, et al. Modelling and analysis of radial distribution network with high penetration of renewable energy considering the time series characteristics. IET Generation Trans & Dist. 2020;14(14):2800–9. doi: 10.1049/iet-gtd.2019.1874

[3] Luo CYL, Li ZW, Xu ZY, et al. Evaluation and analysis of factors influencing the operation and inspection cost of distribution network equipment assets. Electr Power. 2023;56:216–27.

[4] Chen 4 X, Li WP, Li ZY, et al. Prospect on key technology of the XLPE insulation materials and HVDC cables. High Volt Eng. 2020;46:1571–9.

[5] Jia 5 X, Wang XM, Gan WC, et al. Research on calibration of bolt’s axial stress based on acoustoelastic effect. China Meas Test. 2018;44:23–7.

[6] Hu 6 F T, Song XL, Lv L, et al. Experimental research on tightening torque limited value based on commercial bolt torque coefficient. Steel Constr. 2018;33:31–6.

[7] Carlson JE, Lundin P. Measurement of the clamping force applied by load-bearing bolts using a combination of compression and shear ultrasonic waves. 2015 IEEE International Ultrasonics Symposium (IUS), 2015. 1–4. doi: 10.1109/ultsym.2015.0079

[8] Ding 8 P, Li ZH, Zhang LH, et al. Obstacle location and recognition method for an inspection robot based on multi-sensors. Mod Manuf Eng. 2021:36–42.

[9] Li H. Research on insulation puncture clamp fastening bolt torque. East China Electr Power. 2012;40(1):902–5.

[10] Chen K, Lu SY, Jiang SL, et al. Research on torque-speed characteristics of asynchronous motor. Software. 2019;40(201):162–5.

[11] Chen LL, He XD, Chen HH. Stiffness calculation and modal verification of bolted connections based on contact analysis. Foreign Electron Meas Technol. 2022;41(1):45–51.

[12] Feng Y, Wu K, Wu SL. Design and simulation of the electric connection and drainage line device for J-type clamp. Mach Des Manuf. 2024:326–30.

[13] Yang D, Shen PF, Chen GY, et al. Analysis and control method of tightening force of bolt pre-tightening device of high-voltage line drainage clamp. Microcomput Appl. 2022;38(1):95–8.

[14] Eroğlu M, Koç MA, Esen İ. Application of magnetic field to reduce the forced response of steel bridges to high speed train. Int J Mech Sci. 2023;242:108023.

[15] Esen I, Abdelrhmaan AA, Eltaher MA. Free vibration and buckling stability of FG nanobeams exposed to magnetic and thermal fields. Eng Comput. 2022;38(1):3463–82.

[16] Zheng Y, Liu L-C, Qu D, Chen C. Nonlinear postbuckling analysis of magneto-electro-thermo-elastic laminated microbeams based on modified couple stress theory. Applied Mathematical Modelling. 2023;118:89–106. doi: 10.1016/j.apm.2023.01.021

[17] Özmen R, Kılıç R, Esen I. Thermomechanical vibration and buckling response of nonlocal strain gradient porous FG nanobeams subjected to magnetic and thermal fields. Mech Adv Mater Struct. 2024;31(1):834–53.

[18] Ozalp AF, Esen I. Magnetic field effects on the thermomechanical vibration behavior of functionally graded biocompatible material sandwich nanobeams. Mech Adv Mater Struct. 2025;32(2025):459–77.

[19] Butner CM, Adams DE, Foley JR. Experimental investigation of the effects of bolt preload on the dynamic response of a bolted interface. J Appl Mech. 2013;80(011016).

[20] Jiang C, et al. Preload loss of high-strength bolts in friction connections considering corrosion damage and fatigue loading. Eng Fail Anal. 2022;137:106416.

[21] Andrade FTB, et al. Prediction of stress components using the Beltrami-Michell method. J Appl Geophys. 2024;222:105309.

[22] Esen I. Response of a micro-capillary system exposed to a moving mass in magnetic field using nonlocal strain gradient theory. International Journal of Mechanical Sciences. 2020;188:105937. doi: 10.1016/j.ijmecsci.2020.105937

[23] Yıldız T, Esen I. On the effect of the Casimir, van der Waals and electrostatic forces on the thermomechanical buckling of sandwich smart piezo magnetic nanosensor/switch plates. Microsyst Technol. 2025;31(2025):1525–45.

[24] Li F, et al. Electrostrictive effect in ferroelectrics: An alternative approach to improve piezoelectricity. Appl Phys Rev. 2014;1(1):011101.

[25] Esen I, Özarpa C, Eltaher MA. Free vibration of a cracked FG microbeam embedded in an elastic matrix and exposed to magnetic field in a thermal environment. Compos Struct. 2021;261:113552.

[26] Apicella V, Clemente CS, Davino D, Leone D, Visone C. Review of modeling and control of magnetostrictive actuators. Actuators. 2019;8(2):45. doi: 10.3390/act8020045

[27] Keim SM, Guisto JA, Sullivan JB. Environmental thermal stress, Ann. Agric. Environ. Med. 9 (2002). 12088391

[28] Yan LJ, Zhang Y. Calculation of temperature rise of high-current closed busbar. High Volt Eng. 2008;34(2008):201–3.

[29] Shen JF, Zhang C, Liu F. Thermally coupled analysis of thermoelastic field in a thin-walled rotating FGM circular disk. Acta Mater Compos Sin. 2019;36(1):1017–28.

[30] An YH, Li QW, Jiang GN, et al. Thermal aging assessment of centrifugal casting austenitic pipe based on thermoelectric power. South Energy Constr. 2025;12(1):142–50. doi: 10.16516/j.ceec.2024-310

[31] Niffenegger M, Leber HJ. Monitoring the embrittlement of reactor pressure vessel steels by using the Seebeck coefficient. J Nucl Mater. 2009;389:62–7.

[32] Morin P, Nochetto RH, Siebert KG. Convergence of adaptive finite element methods. SIAM Rev. 2002;44(4):631–58. doi: 10.1137/s0036144502409093

[33] SAC/TC 202 NTC 202 on OL of SA of C. Test methods for electric power fittings — Part 1: Mechanical tests: GB/T 2317.1-2008. Beijing: China Standard Press. 2008.

[34] SAC/TC 36 (National Technical Committee 36 on Live Working of Standardization Administration of China), Technical guide for live working on distribution lines: GB/T 18857-2019 [S], China Standard Press, Beijing, 2019.

[35] Wu QB, Ma DE, Zou DH. Simulation analysis of J-type clamp based on multi-physics coupling. Southern Power System Technology. 2021;15(1):115–21. doi: 10.13648/j.cnki.issn1674-0629.2021.05.0014

